# Delivering blended bioinformatics training in resource-limited settings: a case study on the University of Khartoum H3ABioNet node

**DOI:** 10.1093/bib/bbz004

**Published:** 2019-02-15

**Authors:** Azza E Ahmed, Ayah A Awadallah, Mawada Tagelsir, Maram A Suliman, Atheer Eltigani, Hassan Elsafi, Basil D Hamdelnile, Mohamed A Mukhtar, Faisal M Fadlelmola

**Affiliations:** 1 Center for Bioinformatics and Systems Biology, Faculty of Science, University of Khartoum, Khartoum, Sudan; 2 Department of Electrical and Electronic Engineering, Faculty of Engineering, University of Khartoum, Sudan; 3 Department of Zoology, Faculty of Science, University of Khartoum, Khartoum, Sudan; 4 Department of Haematology and Immunohaematology, Faculty of Medical Laboratory Sciences, Ibn Sina University, Khartoum, Sudan; 5 Department of Biology, Faculty of Medicine, Ibn Sina University, Khartoum, Sudan; 6 Department of Medical Biotechnology, Commission for Biotechnology and Genetic Engineering, National Centre for Research, Khartoum, Sudan; 7 Medicinal, Aromatic Plants and Traditional Medicine Research Institute, National Centre for Research, Khartoum, Sudan; 8 Faculty of Pharmacy, Karary University, Omdurman, Sudan

**Keywords:** bioinformatics training, blended learning, bMOOC, distance-based learning, resource-limited settings

## Abstract

**Motivation:**

Delivering high-quality distance-based courses in resource-limited settings is a challenging task. Besides the needed infrastructure and expertise, effective delivery of a bioinformatics course could benefit from hands-on sessions, interactivity and problem-based learning approaches.

**Results:**

In this article, we discuss the challenges and best practices in delivering bioinformatics training in resource-limited settings taking the example of hosting and running a multiple-delivery online course, Introduction to Bioinformatics, that was developed by the H3ABioNet Education and Training working group and delivered in 27 remote classrooms across Africa in 2017. We take the case of the University of Khartoum classrooms. Believing that our local setting is similar to others in less-developed countries, we also reflect upon aspects like classroom environment and recruitment of students to maximize outcomes.

## Introduction

The rapid advancements in genomics and molecular biology research and applications necessitate adequate, up-to-date and complimentary training in biology and computer science [1, 2].

Physical face-to-face bioinformatics training workshops are one way to address this need, by providing opportunities for networking and first-hand discussions [3]. However, in settings of limited access to local bioinformatics expertise, funding or proper infrastructure [4], this model becomes very expensive to run and modest in students’ intake. Of question is the relevancy and applicability of skills acquired from such training to the bioinformatician’s own environment in the long term [5].

Learning methods connecting distant learners and educators have evolved with technology, from postal services to massively open online courses (MOOCs) [6]. For bioinformatics training, both edX and Coursera, two popular MOOC providers, offer complete specializations for both biologists (https://www.edx.org/micromasters/bioinformatics) and computer scientists (https://www.coursera.org/specializations/bioinformatics). Yet, some MOOCs may be inaccessible for developing countries’ learners due to conditions like technological access, digital literacy, cultural relevance and social identity threats [7, 8], along with the typical caveats associated with MOOCs: competing priorities of learners and information overload [9].

H3ABioNet, the pan African Bioinformatics Network [10], is making strides toward bridging the bioinformatics training gap in Africa by designing and offering a 3-month multiple-delivery-mode training course, Introduction to Bioinformatics (IBT), across all its nodes including Sudan [5]. The IBT model blends local face-to-face tutoring sessions with online elements delivered via the course website (https://training.h3abionet.org/IBT_2017/), the online learning management system, Vula (http://www.cilt.uct.ac.za/cilt/vula), and the open-source videoconferencing system, Mconf (https://mconf.sanren.ac.za/).

This study assesses the efficiency and effectiveness of the 2017 iteration of the IBT (IBT_2017) training model from the local learner’s and teaching assistants’ (TAs) perspectives in the H3ABioNet node of Sudan based at the University of Khartoum. We investigated factors contributing to training success, as inferred from responses to surveys disseminated to both local learners and TAs. Our results agree with empirical data suggesting that local group discussions improved students’ ability to access the course materials [11], especially when facilitated by volunteering course alumni [12]. Also, the African context ingrained into the course made the content relevant to the local learners, and hence the training aligned with their expectations [7]. Our setting resembles others in less-developed countries, so we further reflect upon aspects like classroom environment and recruitment of learners to maximize outcomes.

## Relevant literature

The effectiveness of MOOCs in less-developed countries is hindered by barriers of technology and context [7]. Efforts addressing these barriers include +Acumen (https://www.plusacumen.org), which aims at empowering social change and provides MOOCs employing in-video transcripts, culturally diverse case studies and content that is viewable offline and platform agnostic. Consequently, +Acumen attracts participants from a diverse pool of countries, including Afghanistan, Botswana and Sri Lanka [13]. Successful participation from those countries and other top fragile states [14] was also reported in AuthorAID’s offering on scientific research writing, which utilized low-bandwidth friendly format via mainly text-based content and voluntary course alumni as facilitators [12].

Interestingly, psychological research has demonstrated significant improvement in persistence and completion rates of less-developed countries’ learners in MOOCs by brief psychological interventions to lessen social identity threats, like value affirmations and social belonging [8]. Furthermore, studies reporting on the application of the blended MOOC (bMOOC) paradigm, combining online MOOC components with face-to-face sessions, have systematically shown positive educational indicators, even when applied in resource-limited settings [11, 15–17].

Especially for bioinformatics, there are huge, urgent, unmet training needs [1] exacerbated by the breadth of the discipline and its rapid evolution [2], besides the difficulty of curricula design to learners of diverse backgrounds [4], and the shortage in experienced, qualified trainers [1]. Therefore, various international efforts were exerted to bridge this skill gap, from organizations and projects like the GOBLET [18], BD2K [19] and H3ABioNet [20]. These efforts focused on short face-to-face and online courses, which are preferable by researchers in their later career stages [1].

However, for basic bioinformatics users [21], both Coursera and edX provide introductory level training aimed at the molecular biologist level. Tables 1 and 2 compare these offerings with H3ABioNet’s bMOOC in terms of delivery, content, length and recognition. These three courses are comparable in terms of their content, and their quality is unquestionable. However, their accessibility to learners in less-developed countries remains to be investigated. In the rest of the article, we highlight and evaluate elements of the bMOOC run by H3ABioNet, the IBT course in its 2017 iteration, that make it particularly accessible to learners in less-developed countries.

**Table 1:** Overall comparison between online bioinformatics courses targeting basic bioinformatics users

Course	IBT_2017^a^	DNA Sequences: Alignment and Analysis (DS:AA)^b^	Bioinformatics Methods – 1(BM1)^c^
Provider	H3ABioNet (African universities)^d^	edX (University of Maryland, USA)	Coursera (University of Toronto, Canada)
Delivery	bMOOC: Instructor-paced with local tutors	xMOOC: Online only, instructor-paced	xMOOC: Online only, instructor-paced
Content	Videos and readings	Largely textual	Videos and readings
Duration (weeks)	13	8	8
Recognition	Statement of accomplishment	Verified certificate^e^	Verified certificate with no credit^e^

^a^https://training.h3abionet.org/IBT_2017/

^b^https://www.edx.org/course/dna-sequences-alignments-analysis-usmx-university-maryland-university-bif001x

^c^https://www.coursera.org/learn/bioinformatics-methods-1

^d^All IBT_2017 trainers are based in African universities except for a guest introductory lecture by a European bioinformatician

^e^A verified certificate is offered at a cost, though financial aid is possible. Without a payment, edX offers a non-verified statement of accomplishment (i.e. honor code certificate), while Coursera restricts access to some parts of the course (and, hence, no statement of accomplishment).

**Table 2:** Content-wise comparison between online bioinformatics courses targeting basic bioinformatics users

Course	IBT_2017	DS:AA	BM1
Genetics review	−	$+$	−
Databases	$+$	$+$	$+$
Linux	$+$	−	−
Sequence similarity	$+$	$+$	$+$
Genomics	$+$	$+$	$+$
Phylogenetics	$+$	$+$	$+$
Gene expression	Extra material	$+$	$+$
Protein structure	−	−	−
Selection analysis	−	−	$+$
Metagenomics	−	−	$+$

IBT_2017: The IBT course from H3ABioNet in its 2017 iteration. DS:AA: The DNA Sequences: Alignment and Analysis course from edX. BM1: The Bioinformatics Methods–1 course from Coursera.

## Methods

### The local settings of the 2017 iteration of the IBT

In its 2017 iteration, the IBT course started on 9 May, with 2 days/week for face-to-face tutorial sessions. Building on its 1st iteration of 2016 [5], the IBT_2017 was composed of six modules: introduction to databases and resources, Linux, sequence alignment theory and application, multiple sequence alignment, genomics, molecular evolution and phylogenetics (Table 2). The design, learning objectives and contents of these modules are already described in [5], so here we only comment on the local supporting set up of the classrooms.

**Figure 1:**
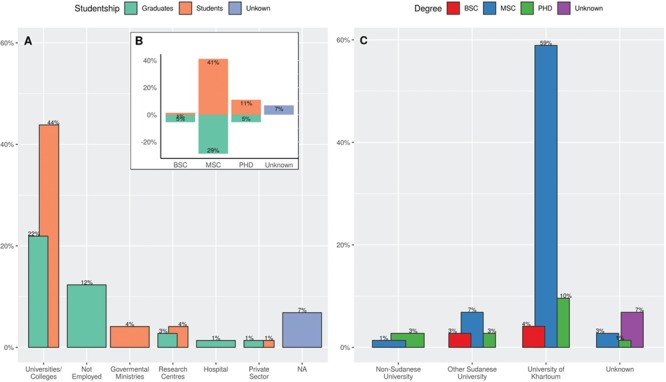
Demographics of the IBT_2017 participants in the H3ABioNet University of Khartoum node, Sudan. (**A**) Different affiliations of IBT participants stratified by their status as current students or graduates. Grouping into the shown categories was done by manual assignment of data to the appropriate category. (**B**) The distribution of the highest academic degree of the IBT participants stratified by their status as current students or graduates. (**C**) The distribution of the institutes awarding the highest degree for the course participants.

In the H3ABioNet node of Sudan, 73 participants enrolled in the IBT_2017. The majority of these participants (or learners herein) have been selected from a waiting list from the IBT_2016 iteration, based on their interest and basic understanding of the central dogma of molecular biology, and hence they came from diverse specializations (Figure 1; Figure SF3), at different educational levels (6% were at the BSc level, 41% current MSc students, 11% current PhD students and 34% were MSc and PhD graduates not pursuing any degree) and career affiliations (66% in academic institutes, 4% in governmental ministries, 7% in research centers, 3% in private companies and hospitals, with 12% unemployment rate) as shown in Figure 1(B and A, respectively). Figure 1C further shows that the highest educational institute for the majority of participants is the University of Khartoum (73%, compared to 13% from the other Sudanese universities and 4% who studied abroad).

Two classrooms were set up for physical face-to-face tutorial sessions within University of Khartoum main campus: the Centre for Bioinformatics and Systems Biology (CBSB) laboratory, equipped with 20 PCs and network ports to accommodate an additional 12 PCs/laptops, and the main library computer laboratory that can accommodate up to 70 participants. Hosting 33 and 40 participants, respectively, these two locations varied in their infrastructure as well: the PCs in the CBSB laboratory are appropriate for bioinformatics training and research with larger screen sizes and more CPU and memory capacities, while those in the main library needed more effort from the local IBT team and University of Khartoum Information Technology Network Administration (ITNA) staff to set them up.

This larger intake (compared with 22 participants in 2016) has been managed by a local staff, which, besides the node principle investigator, was composed of 7 TAs who are among the alumni of the IBT_2016 with MSc-level degrees in genetics and molecular biology. Their prior IBT experience helped them to provide actionable support to the IBT_2017 participants, and their facilitation job was eased with the online staff training sessions provided by the IBT core team. Only a single system administrator was available for the duration of the IBT_2017, given the physical proximity of the local classrooms.

### Measurement and evaluation

**Figure 2:**
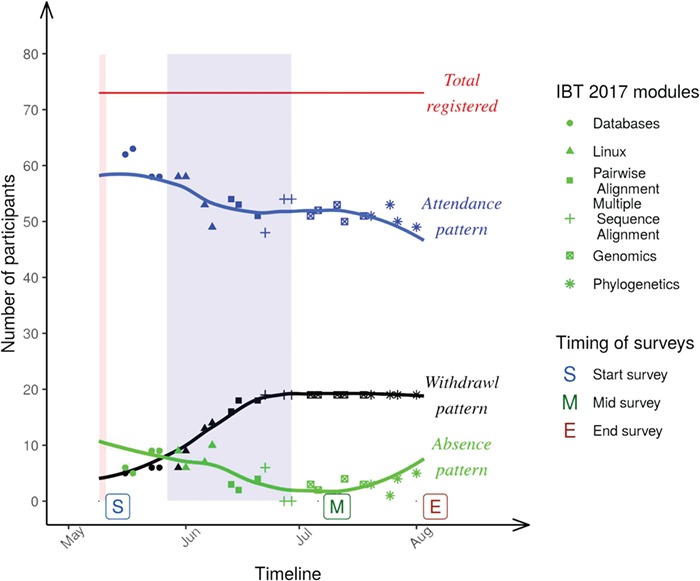
Patterns of attendance, absence and withdrawal in the six IBT_2017 modules in the H3ABioNet University of Khartoum node, Sudan. Points of data collection (surveys) are also highlighted in the timeline. The red-shaded area coincides with the orientation week, whereas the blue-shaded area is the holy month of Ramadan.

While we lack data on the performance details of our IBT_2017 participants (individual assignments and assessments were only managed by the IBT core team in South Africa), we have alternative aggregate data from the following sources:

i. The waiting list from the previous IBT_2016, containing demographic data on our course participants.
ii. Participants’ attendance in the face-to-face tutorial sessions (Figure 2), along with their experience throughout the course inferred from three surveys distributed at the start, middle and end points of the course (Supplementary SM1, SM2 and SM3, respectively).
iii. There are also data identifying participants who earned a certificate upon successfully satisfying the course requirements.
iv. An exit survey was also designed and disseminated to withdrawn course participants to capture the reasons motivating their withdrawal (Supplementary SM4).
v. Another survey was designed and disseminated to the local TAs, assessing the extent to which the course experience was valuable to them (Supplementary SM5).
vi. Finally, a follow-up survey was sent to the course participants 9 months upon their completion of the IBT_2017 (Supplementary SM6).

Collectively, we used this data to investigate factors associated with course completion, failure or withdrawal, so that we are better informed for future course runs or similar training initiatives.

## Results

### Retention and classrooms demographics

Unlike face-to-face courses, MOOCs generally have both high enrollment rates and staggering low completion rates [9, 22, 23]. This attrition rate is typically altered to learners’ intentions to focus on specific parts of a given MOOC and not to fully complete it [24] or their struggle in committing the needed study hours into their schedules [9].

In our 3-month bMOOC IBT_2017, course completion means successful satisfaction of all the course requirements: physical attendance in the face-to-face tutorial sessions, submission of at least 90% of the practical assignments following each contact session (through Vula) and a mark of at least 60% in the assessment exercises following each module (also through Vula).

Attendance-wise, we note that for our local 73 enrolled participants, the attendance rate was higher at the beginning of the course (∼85%), then it dropped progressively toward mid-June and early July (Figure 2), concurrent with gradual increase in withdrawal. Additionally, looking at the absence pattern in Figure 2, we also note that missing a given IBT session does not necessarily mean total withdrawal from the course in our case, as sometimes learners did manage to work toward their practical assignments and assessments despite missing the face-to-face session (though we lack details on individual students’ performance in these aspects).

Poor response rate (only 1) from withdrawn participants to the exit survey (Supplementary SM4) limits our ability to conclusively reason about drop-out motives, except to note from Figure 2 that (1) sharp drop out co-occurrs with the Linux module, which requires a mode of thinking a bit alien to wet-lab biologists; (2) culturally, the IBT_2017 started just a few weeks before the holy month of Ramadan, coinciding with end-of-year/semester holidays in many universities and colleges (full- and part-time students make up 44% and 9% of the total IBT_2017 participants, while other academic staff makes up 22%). However, once these holidays were over, academic participants (collectively, 75%) needed to be back to full-time working hours and classes in their respective institutes, making it harder for them to attend IBT_2017 sessions in person and timely work toward their assignments and assessments. Figure 2 further shows that the withdrawal pattern plateaued after this point in time, totaling 5 and 14 participants from the CBSB and main library classrooms, respectively, summing to 19 participants (26% of the total).

Remarkably, Figure 1C shows that the majority of the IBT_2017 participants (73%) have graduated from University of Khartoum, as did the local course staff. This could justify why ∼63.6% of the participants heard of the course through their friends, 27.3% from their supervisors or mentors and the remaining 14.5% through social media (Supplementary SF1). It also suggests that local circles of friends/acquaintances were already in place before the course had actually started. This support system in place could explain why out of the 54 participants who did not withdraw (∼74% of the total), only 3 (4% of the total) failed the course requirements.

### Participants’ perceptions and expectations

The experience of the IBT_2017 is both unique and new to our participants considering its blended multi-delivery learning model [5] and its extended 3 months duration. Our three surveys (Supplementary SM1, SM2 and SM3) disseminated at the start of the course, the mid-point and the end (Figure 2) aimed to check the alignment between participants’ expectations and course scope [25]. On a labeled five-point Likert scale, we asked participants about their prior experience level in each module (start survey); the extent to which the content was appropriate and the level it met their expectations (in mid-point and end surveys, for each module taught until that point).

**Figure 3:**
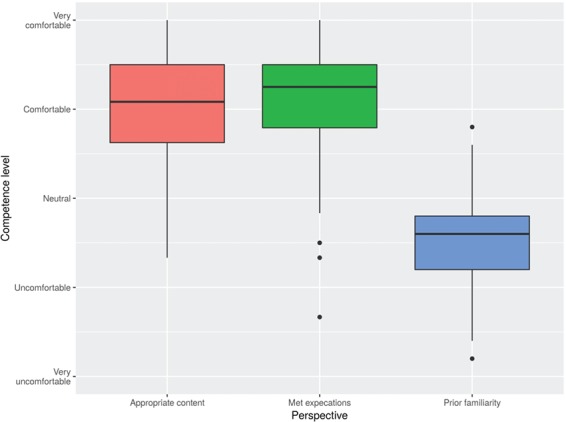
Average perceptions from H3ABioNet University of Khartoum node participants in the IBT_2017 on the six taught modules, in terms of their level of competence, measured as the level of their prior familiarity with the modules, and their satisfaction upon completion according to the responses collected via the start, mid-course and end surveys.

Participants’ perceptions on each of the six modules largely followed the same trend (Supplementary SF7–SF12); hence, their average across all modules is presented in Figure 3. Not surprisingly, most participants were initially largely unfamiliar with the various modules (with the 75th percentile of responses below neutral familiarity level), especially Linux (Supplementary SF8). Progressively, however, we see higher satisfaction levels (with the median of the averaged responses at a level above comfortable in Figure 3) in terms of appropriateness of the taught material and meeting participants expectations.

**Figure 4:**
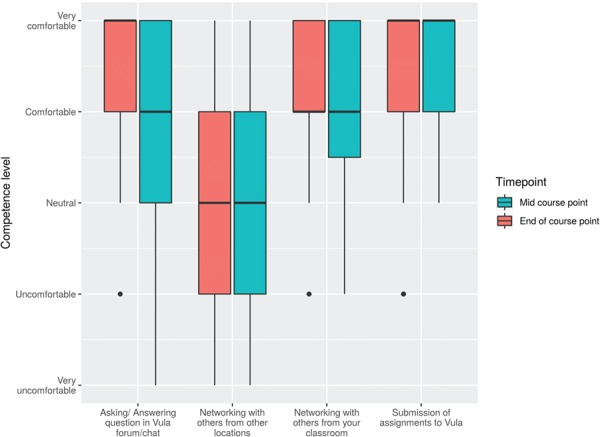
H3ABioNet University of Khartoum node participants’ in the IBT_2017 utilization levels of various elements of online learning: Vula and networking measurements were taken from surveys in the middle and end of the course.

Another aspect investigated is the extent to which our participants made use of the local and remote classroom elements to ameliorate their learning experience. Namely, we were interested in networking with others and the use of Vula. We monitored the progression in these aspects at the mid-course and end surveys. The responses depicted in Figure 4 show a positive trend as the course advanced, suggesting more familiarity with the bMOOC model. Yet, we remark a modest and steady amount of networking with other IBT classrooms throughout the course [In total, 38 participants filled both the mid-course and end surveys, with 2 and 5 unique responses for each survey respectively (Supplementary SF2)].

## Discussion

### Retention and classrooms demographics

Given its blended nature, the IBT_2017 required both physical attendance and online, timely submission of assignments and assessments for successful completion of the course. The plateau in the withdrawal pattern of Figure 2 is a bit at odds with the expectation in typical MOOCs, where enrolled participants tend to continue dropping throughout the course [23, 24]. While we mainly alter this withdrawal to cultural circumstances in our bMOOC (Retention and classrooms demographics), we remark that drop out is generally lower in bMOOCs than MOOCs [26], since the face-to-face investment of time and resources makes it easier for participants to decide on their commitments realistically when they initially enroll.

Overall, there is a high degree of class imbalance in the data set of our participants in both University of Khartoum classrooms (70% successful completion, 4% failure and 26% withdrawal rates), even when seen in each classroom independently (85%, 0% and 15% for the CBSB class, and 58%, 8% and 35% for the main library, respectively). Additionally, the small sample size of each classroom taken alone also prevents a meaningful statistical analysis applied to each classroom alone. We therefore limit discussions here to key demographic factors derived from a multinomial classification model applied to the complete data set (both classrooms at once) (Supplementary Figure SF4, ST1 and Listing2). Acknowledging this models’ assumptions of independence and constant performance, we still see that both the physical location of the classroom and gender are the only statistically significant predictive factors, with a note that MSc and PhD participants had higher odds of success than BSc level candidates, as did unemployed participants and those working in research centers or the private sector. Whether a participant is currently a student or has graduated from the said level had slight effect on their odds of success.

The location of the local IBT_2017 classroom (the CBSB laboratory or main library) is effective in predicting performance (Supplementary SF3, SF4 and SF6). This is expected considering the inherent infrastructure differences between the two locations; the CBSB laboratory is designed to facilitate bioinformatics training and research in terms of stable internet connection and more powerful computers, whereas the main library classroom had Internet connectivity issues at the beginning of the course, which was frustrating to some of the participants (and, in occasions, encouraged some to withdraw early on).

Consistent with the largely female student body in faculties related to natural sciences, health and agriculture in Sudan [27], the majority of the IBT_2017 participants in both classes of the H3ABioNet node of Sudan were females (80%) and they were also more likely to satisfy the course requirements in comparison with their male peers (Supplementary SF3–SF5).

Figure 1A underscores that about half the entire local IBT_2017 classes (53%) are students, 15% of whom are part-timers with affiliations in either governmental ministries or research laboratories. We reckon the better odds of success for students to their interest in a specific problem at hand. We see no statistical evidence of interaction between part/full-time status and studentship; hence, it is not shown in the ST1 model. However, the ability of part-timers to satisfy the course requirements and acquire higher odds of success (Supplementary SF3 and SF4) suggest the appreciation of these institutes to staff training and possibly hints at avenues for local sustainable research collaborations.

**Figure 5:**
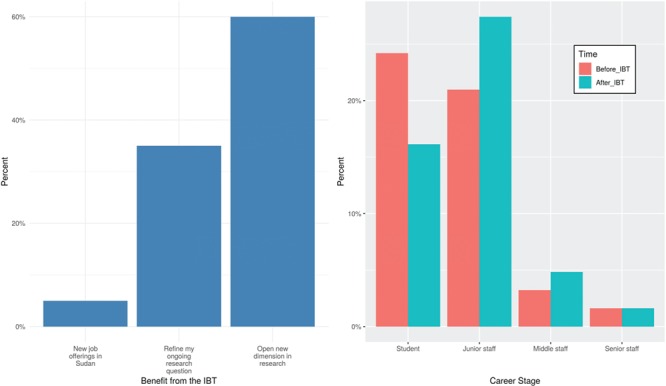
Feedback from the IBT_2017 alumni from the H3ABioNet University of Khartoum node taken after 1 year. **(A)** Benefit gained from the IBT_2017 training. **(B)** Career progression between the starting time of the IBT_2017 and 1 year later.

Yet, there is 12% unemployment ratio among our participants upon starting the IBT_2017. While we did not explicitly investigate the employability of typical biological sciences graduates in Sudan, by large, our participants indicated that their pursuit of graduate education was motivated by hopes in better career opportunities. For our participants successfully completing the IBT_2017, the feedback that was collected 9 months upon the course end (filled by 31/51 of those successful participants) indicates that many of the then-students participants have completed their degrees and were offered jobs based on their new skills (Figure 5B). Yet, we see that the biggest value of the IBT_2017 has been more invisible as it helped those participants refine their research interest (Figure 5A). The same employability pattern was observed with the IBT_2016 MSc and PhD alumni who received offers of teaching positions in local newly established faculties to teach bioinformatics-related courses or computational laboratories (personal communications). This is in line with recent literature supporting improved job salary and mobility upon completion of MOOCs [28]. Yet, it remains inconclusive that graduating the IBT_2017 alone was the factor enhancing our participants’ employability.

### Participants’ perceptions and expectations

By design, the IBT course is taught over 3 months, to train basic bionformatics users. Besides providing relevant and high-standard content, the IBT core team and various instructors have placed great emphasis on defining clear learning objectives and outcomes prior to each taught module and maintained the logical structure of the course despite removing the structural proteomics module in the IBT_2017 [5].

Therefore, course participants had clear expectations out of each module, manifested as largely positive indicators in their responses to our surveys (Figure 3; Supplementary SF7–SF12), and high success rate (excluding early withdrawals, 94% of the participants satisfied the course requirements, see also Retention and classrooms demographics). Considering that the majority of the participants were current graduate students (Figure 1B; Supplementary SF3) in a domain related to genetics or molecular biology (Supplementary SM8), they were exposed to concepts and uses of databases but less familiar with Linux, Genomics and related topics because there are none or limited postgraduate degrees in bioinformatics currently in Sudan.

Regarding utilizing local and remote classroom resources, we make few observations. While we see that our participants were comfortable networking with each other, we see less interactions with participants from other classrooms (Figure 4). Partially, this is attributable to the demographics of the classrooms (Retention and classrooms demographics). While a few minutes were spared at the beginning of each session for the scattered classrooms to introduce themselves via Mconf, this was ineffective in linking our participants with other classrooms (Figure 4) because often times the connection would be too noisy for a meaningful conversation. The same issue often arose during the discussion time at the end of each session when the instructors are typically available and answering questions live. Alternatively, our participants tended to discuss issues through the forum or the chat rooms available through Vula. There are more engagements in these avenues toward the end of the course (Figure 4).

Another element explaining the high completion rate of the two classrooms in the University of Khartoum is the local help provided to the participants through the local TAs who were IBT_2016 alumni. From the literature, testimonials from successful alumni have been shown to improve the sense of belonging in MOOCs and, thereby, learners’ performance [8]. This is especially true as the IBT_2017 included training sessions for the local team in each classroom on how to best facilitate the course. These training sessions employed the mental contrasting with implementation intentions model [29] in equipping the local staff with best strategies for course facilitation by asking them to set goals for the sessions and then predict future challenges and devise contingency plans. While not all identified challenges were within direct control (Reflections on logistics), the exercise gave a sense of confidence to the local staff. Additionally, the majority of our volunteering staff already had prior teaching experience (7/8 TAs, in addition to the class PI). In a typical tutorial session, they would be timing the videos for streaming, promoting within-classroom discussions with course participants or explaining new concepts drawing from their own experiences. Typically, they would not restrict themselves to purely using English in these discussions. After videos, they would still be available to help students using the learning management system, Vula, in submitting assignments or assessments or using the forum or chat room. A weekly meeting was set up with local staff to go through problems faced the previous week or requirements needed for the upcoming one. Additionally, there were meetings with the IBT core team to follow on progress and issues faced. The responses from those TAs upon IBT_2017 end indicate that they benefited from their previous experience, so they were comfortable facilitating demographically diverse classes (Supplementary SF13). On the personal level, the TAs were highly satisfied with the experience, even though they had other pressing commitments playing at the same time of the IBT_2017 (Supplementary SF14).

A final element in examining the IBT_2017 is its non-cognitive factors. These include the expected load incurred by participating in the IBT, which many found to be overwhelming when compared with commercial 1 week bioinformatics courses with no assignments or assessments. This perception has exacerbated especially with the Linux module as can be seen in Figure 2. Aside from the cultural context examined in section (Retention andclassrooms demographics), another notable non-cognitive factor to consider is the course language. Sudan is officially an Arabic speaking country, though university educated graduates have some competency in English. However, one teaching style commonly employed, especially at the undergraduate level, is to deliver teaching curricula in Arabic, while maintaining the English keywords and having English-based handouts (Supplementary SF15). This means that for many of the participants, complete course delivery in English is challenging. Collectively, these factors could explain the large withdrawal from the course before the mid-point (26% of the total participants; Figure 2). A possible workaround might be for the course participants to take a preliminary scientific English course, either in a professional center in Sudan or via MOOCs, like the positively reviewed Coursera offering of ‘English for Science, Technology, Engineering, and Mathematics’ available on (https://www.coursera.org/learn/stem).

### Reflections on logistics

The IBT_2017 is a blended [15] or hybrid [17] MOOC employing a multi-delivery learning model [5].

The efficiency of such models has been reported from studies comparing bMOOCs with face-to-face courses taking place in different countries and targeting diverse skills (USA, electronics and circuits [15], Egypt, teaching methodologies [11] and Rwanda: gaming, entrepreneurship and e-learning [16]). Unlike these works, the IBT model tries to bridge the knowledge gap between current offerings of formal MSc courses (especially in African universities) and the skills and knowledge needed for those students to do bioinformatics projects and research in Africa. Understandably, replicating a 3 months course in more than 1 location is challenging due to appreciable cost restrictions, and, hence, a comprehensive comparison with a 3 months face-to-face course run in other locations is challenging to make.

Compared with other online only MOOCs, like those cited in Table 1, the IBT_2017 model capitalizes on three elements: (1) the use of a multitude of delivery modes (online forums, video conferencing, learning management system, Open Educational Resources (OER) and pre-recorded lectures) allowing efficient use of Internet bandwidth when one delivery mode augments (or even replaces) the other [5]. The online resources used, Mconf for example, were open source and were not network intensive, which made them appropriate to the local set up. Additionally, lecture videos containing the instructor face made the content more lively and engaging [30] than the edX’s mostly textual offering (DS:SS). (2) The geographical distribution of almost all course instructors across African universities provided a familiar context for the participants to relate to [7, 8]. (3) The support by local TAs who are course alumni [31, 32]. Beyond these aspects, we are limited in power to compare with students’ performance on the offerings from edX and Coursera, but it is interesting to pursue such evaluations in the future.

By large, one can see that the design and running of the IBT_2017 followed the 10 simple rules for developing a short bioinformatics training course of [25]. Particularly, we comment on the application of the following rules:

- **Setting practical and realistic expectations—achieved by specifying target audience (Rule 1, [25])**: The target audience for the IBT are participants with molecular biology background. Those were the participants able to satisfy the course requirements, while graduates from certain faculties (like computer science) withdrew early on. Otherwise, in our case, it did help in yielding a high success rate that we had a long waiting list in place.
- **Class diversity:** In terms of academic backgrounds, graduating universities and career stage (Figure 1; Supplementary SF3). Additionally, proper advertising and conscious selection of participants (in lights of the drop out factors we highlighted in Retention and classrooms demographics) should help in a better educational outcome.
- **Ensuring computational equipment preparedness and support availability (Rule 3, [25])**: Before and throughout the IBT, the local IBT_2017 team would meet weekly and update on resources needed and tasks assigned. A myriad of platforms were used for this: Trello boards for planning and follow up (https://trello.com/), a Google mailing list for emails and Authorea (https://www.authorea.com) for collaboratively working on this manuscript. For some of the members, a chatting app like Whatsapp (https://www.whatsapp.com/) was needed, but, overall, experiencing other platforms was deeply appreciated and highly regarded.
- **Allowing interactivity and providing time for reflection, individual analysis and exploration (Rule 9, [25])**: This was achieved via the in-person sessions, with some of the IBT_2016 alumni as TAs. Those sessions assured accessible local support to the IBT_2017 participants and thereby reduced their anxiety, which is otherwise a major MOOC withdrawal reason [9]. Also, for learners from less-developed countries, success stories from previous course alumni have been shown to improve performance [29]
- **Career progress and capacity building**: The IBT aims to equip African researchers with the skills and knowledge to launch their careers and establish their science. The 31 responses from the follow-up survey that was sent 9 months upon the end of the IBT_2017 show that many participants have moved from being students (53.3% at the time of the IBT) to being junior or middle staff (collectively 56.3%; Figure 5B). For some of them, the IBT helped in securing new job offerings (Figure 5A).

## Challenges and lessons learnt

Design-wise, the IBT_2017 benefited from clearly defined learning outcomes, interactive tutorial sessions between trainers and course participants and sufficient time for hands-on sessions facilitated by local TAs. Collectively, these elements gave participants clear expectations, and they were able to both evaluate their progress and meet the course requirements. The practical face-to-face sessions were particularly important, as trainees often fail to appreciate the applicability of theoretical material to real data. Additionally, a variety of online resources were used for the IBT_2017 delivery. Particularly, a learning management system, Vula, was utilized to send out announcements, manage participants, track progress and allow offline interactions among participants, trainers and staff. Online interactions took place via Mconf (https://mconf.sanren.ac.za/), an open-source videoconferencing system. Mconf features were mostly satisfactory (real-time chat, camera feed, screen sharing, file sharing and classroom mode). Issues with sound clarity and disconnection motivated the IBT core team to consider alternative platforms in the 2018 IBT iteration (personal communication).

The local settings in the University of Khartoum classrooms employed factors that complemented the IBT_2017 online design elements. For example, taking IBT_2016 alumni as volunteering TAs improved the experience of the IBT_2017 participants and was later on adopted by other African nodes hosting subsequent IBT iterations. Also, compared to the IBT_2016 course intake of only 22 participants, we were successful to intake 73 participants for the IBT_2017. This increase, ∼231%, is altered to 3 factors: (1) the actual local need for the provided knowledge and skill, (2) the good words spread about the IBT_2016 from its participants that generated a lot of momentum and interest in subsequent iterations. Following the same trend, there is more than 400 pre-registered individuals for the IBT_2018, coming from different universities, career and educational backgrounds. This diversity is sought as it contributes to a more interactive classroom setting, where learners help each other and build on each others’ complimentary skills. (3) The effective partnership with the University of Khartoum main library, which allowed hosting more participants than the CBSB laboratory capacity.

The main challenges in organizing blended courses like the IBT are the location and timing logistics. The long duration of this course, 3 months, is bound to overlap with parts of the academic year in most of the relevant faculties in a university, and, hence, those faculties cannot offer their properly set up laboratories, which ultimately limits the intake of participants. Timing is also challenging both to the unique cultural circumstances in each classroom, like the various national and religious holidays, and also because some of the course participants could be full-time students with some of the IBT sessions colliding with the timing of their classrooms or exams (∼25 participants in our case). The flexibility and sensitivity of the IBT core team in giving some grace period help those participants make up for missed activities. Additionally, working closely with concerned entities within the university and other governmental entities, like the Ministry of Higher Education, provides support in allocating more infrastructure and resources and also helps sustaining the effort. Such collaborations are especially needed with other universities/research centers besides Khartoum for broader impact. The success of the IBT_2017 experience is a motivator for arranging similar courses (bMOOCs) in other areas like data science and health informatics.

## Author contributions

- Conceptualization: A.E.A., F.M.F.
- Formal analysis: A.E.A., F.M.F.
- Funding acquisition: F.M.F.
- Investigation: A.E.A., A.A.A., M.T., M.A.S., A.E., H.M.E., B.D.H., M.A.M., F.M.F.
- Methodology: A.E.A., A.A.A., M.T., M.A.S., A.E., H.M.E., B.D.H., M.A.M., F.M.F.
- Project administration: F.M.F.
- Resources: F.M.F.
- Software: A.E.A.
- Supervision: F.M.F.
- Writing/original draft: A.E.A., A.A.A., F.M.F., M.A.S., M.T., H.M.E.
- Writing/review and editing: A.E.A., A.A.A., M.T., M.A.S., A.E., H.M.E., B.D.H., M.A.M., F.M.F.

## Supplementary Material

Suppl_bbz004

## Notes

Key Points

- Learners in less-developed countries are keen on seizing educational opportunities. For MOOCs to be attractive, some effort is needed.
- Blended learning employing multi-delivery models can be effective in bridging the achievement gap in MOOCs, even in bioinformatics courses.
- The recruitment of local TAs from course alumni improves the sense of belonging to a MOOC and, hence, improves learners’ performance.
- Working in tandem with other local bodies to secure the needed infrastructural resources is another factor contributing to a successful training.
