# Supplementary material for: Delivering blended bioinformatics training in resource-limited settings: a case study on the University of Khartoum H3ABioNet node
Source: Brief Bioinform. 2019 Feb 15;21(2):719–28. doi: 10.1093/bib/bbz004 (PMC7299290; doi:10.1093/bib/bbz004)
Supplement: Suppl_bbz004 [file suppl_bbz004.zip › Figures_and_models.pdf]

Delivering Blended Bioinformatics Training in Resource-Limited Settings

A case study on the University of Khartoum H3ABioNet Node

Supplementary materials

December 12, 2018

Listings

1 rpart tree model 2
2 10 fold multinomial model validation 3

List of Figures

SF1 **Media effect:** How participants knew about the IBT 5
SF2 **Surveys filled by participants** Consistency of filling the 3 evaluation surveys by the IBT participants in Sudan node: Main library & CBSB laboratory. The numbers represents how many participant filled the given survey from the total 73. For example, 33 filled all surveys, and 15 filled no survey at all, and so on. . . 6
SF3 **Class demographics and performance distribution:** A) Generalized pairs plot of the logistics and demographics of the class (lab location, Gender, Educational level and Studentship) against IBT participants' performance (Success, Withdrawal or Failure). B) Participants' Participants' affiliations distribution with respect to their performance 7
SF4 **10-fold cross validated multinomial model of participants' performance** (Success, Withdrawal or Failure) based on the logistics and demographics of the H3ABioNet University of Khartoum Node classes (lab location, Gender, Educational level and Studentship). Mathematical model is provided in Supplementary Table ST1 7

SF5 **rpart performance classification tree:** Recursive PARTitioning (rpart) classification model of the main covariates affecting participants performance in the IBT (Success, Withdrawal, or Failure). Each node shows the predicted learner performance, the probability of each performance category based on the node group, and the percentage of observations in the node. Here, we see that Gender is most important covariate for predicting the performance of an IBT course participant as per the 2017 data. 8
SF6 **Conditional Inference Tree for Participants Performance in the IBT.** Here, we se that the physical classroom location is the most important covariate in predicting an IBT participant's performance 8
SF7 IBT participants perceptions: Databases module 9
SF8 IBT participants perceptions: Linux module 9
SF9 IBT participants perceptions: Pair wise alignment module. For the prior familiarity, we used the same responses in producing figure SF10 , because participants were expected to have low familiarity with both types 9
SF10 IBT participants perceptions: multiple sequence alignment module. For the prior familiarity, we used the same responses in producing figure SF9 , because participants were expected to have low familiarity with both types 10
SF11 IBT participants perceptions: Genomics module 10
SF12 IBT participants perceptions: phylogenetics module 10
SF13 TAs perspective: Evaluation of the teaching experience (n =7) . 11
SF14 TAs perspectives: Personal reflections (n=7) 11
SF15 Language of instruction of the IBT participants in their BSc and MSc education 11

1 Mathematical models details

Listing 1: rpart tree model

```
Call:
rpart(formula = pass ~ lab + Gender + educational_level + Studentship
    +
Affiliations, data = data_grads_demographics, method = "class")
n= 73

CP nsplit rel error xerror     xstd
1 0.04545455     0 1.0000000 1.000000 0.1782019
2 0.01000000     1 0.9545455 1.090909 0.1824398

Variable importance
Gender
100

Node number 1: 73 observations, complexity param=0.04545455
predicted class=Success expected loss=0.3013699 P(node) =1
```

```
class counts:   51    19    3
probabilities: 0.699 0.260 0.041
left son=2 (59 obs) right son=3 (14 obs)
Primary splits:
Gender          splits as LR,    improve=2.2747350, (0 missing)
lab             splits as LR,    improve=2.1665210, (0 missing)
Affiliations    splits as RRLLLR, improve=1.3764880, (9 missing)
Studentship     splits as LRL,   improve=0.8760305, (0 missing)
educational_level splits as RLRL, improve=0.2866640, (0 missing)

Node number 2: 59 observations
predicted class=Success expected loss=0.2372881 P(node) =0.8082192
class counts:   45    12    2
probabilities: 0.763 0.203 0.034

Node number 3: 14 observations
predicted class=Withdraw expected loss=0.5 P(node) =0.1917808
class counts:    6     7    1
probabilities: 0.429 0.500 0.071
```

Listing 2: 10 fold multinomial model validation

```
Penalized Multinomial Regression

64 samples
5 predictors
3 classes: 'Success', 'Withdraw', 'Fail'

No pre-processing
Resampling: Cross-Validated (10 fold)
Summary of sample sizes: 57, 58, 58, 57, 57, 58, ...
Resampling results across tuning parameters:

decay  Accuracy  Kappa
0e+00  0.6591667 0.07751040
1e-04  0.6591667 0.07751040
1e-01  0.7401190 0.08571429

Accuracy was used to select the optimal model using the largest value.
The final value used for the model was decay = 0.1.
```

Table ST1: 10-fold cross validated multinomial model of the IBT 2017 participants' performance in the course as measured in the H3ABioNet node of Sudan

	Dependent variable:	
	Withdraw	Fail
	(1)	(2)
labMain_Library	1.103*	1.206
	(0.642)	(1.361)
GenderMale	1.374*	0.883
	(0.721)	(1.510)
educational_levelMSC	−1.362	−0.394
	(1.546)	(2.914)
educational_levelPHD	−1.029	−1.243
	(1.676)	(3.576)
StudentshipStudents	0.094	0.999
	(0.697)	(1.767)
AffiliationsHospital	1.596	−0.190
	(2.772)	(5.975)
AffiliationsNot_Employed	−1.580	−1.344
	(2.206)	(3.421)
AffiliationsPrivate_Sector	−0.993	−0.808
	(2.825)	(3.127)
AffiliationsResearch_Centres	−1.030	−1.386
	(1.938)	(2.857)
AffiliationsUniversities_Colleges	−0.043	−1.149
	(1.534)	(1.805)
Constant	−0.452	−2.624
	(2.304)	(3.893)
Akaike Inf. Crit.	131.217	131.217
Note:	*p<0.1; **p<0.05; ***p<0.01	

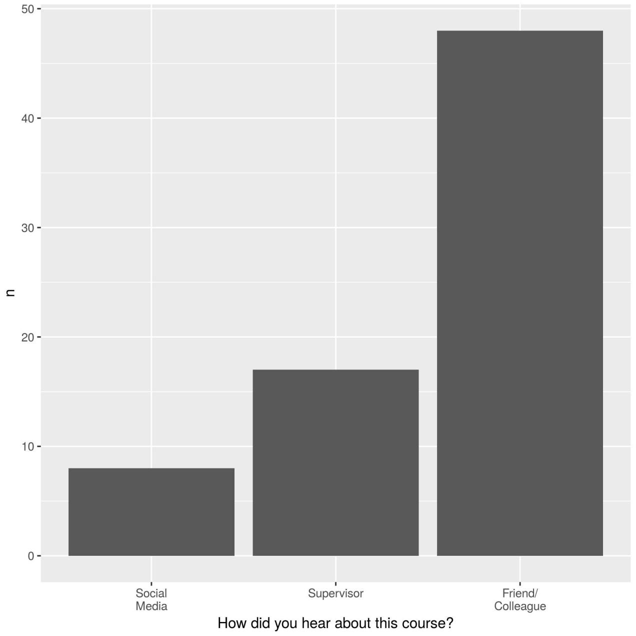

Figure SF1: **Media effect:** How participants knew about the IBT

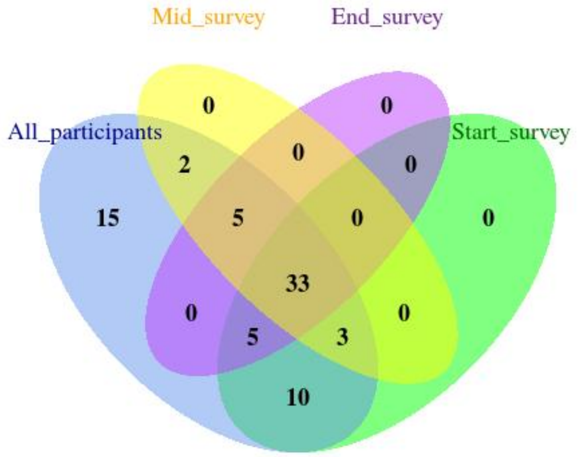

Figure SF2: **Surveys filled by participants** Consistency of filling the 3 evaluation surveys by the IBT participants in Sudan node: Main library & CBSB laboratory. The numbers represents how many participant filled the given survey from the total 73. For example, 33 filled all surveys, and 15 filled no survey at all, and so on.

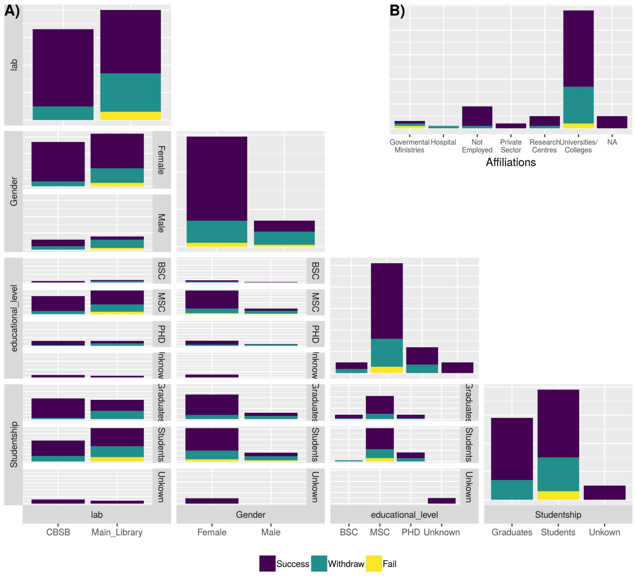

Figure SF3: **Class demographics and performance distribution:** A) Generalized pairs plot of the logistics and demographics of the class (lab location, Gender, Educational level and Studentship) against IBT participants' performance (Success, Withdrawal or Failure). B) Participants' Participants' affiliations distribution with respect to their performance

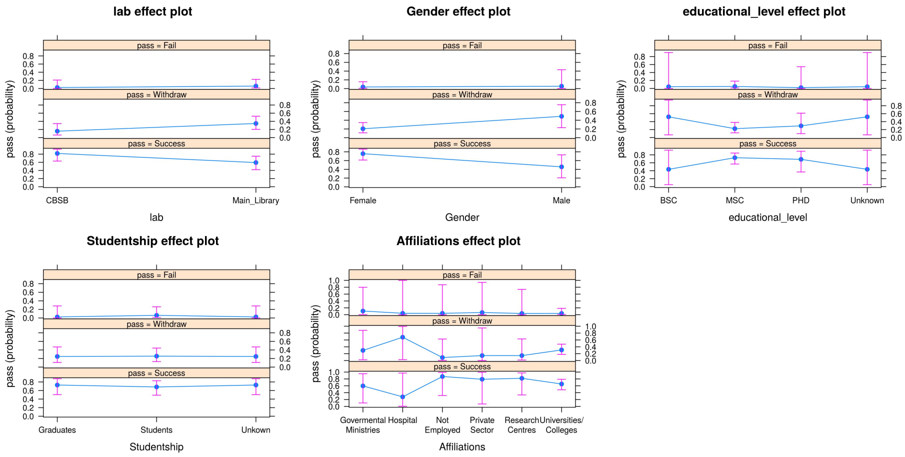

Figure SF4: **10-fold cross validated multinomial model of participants' performance** (Success, Withdrawal or Failure) based on the logistics and demographics of the H3ABioNet University of Khartoum Node classes (lab location, Gender, Educational level and Studentship). Mathematical model is provided in Supplementary Table ST1

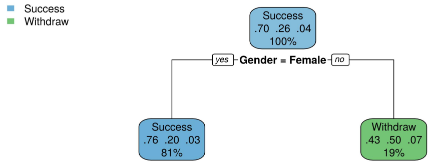

Figure SF5: **rpart performance classification tree:** Recursive PARTitioning (rpart) classification model of the main covariates affecting participants performance in the IBT (Success, Withdrawal, or Failure). Each node shows the predicted learner performance, the probability of each performance category based on the node group, and the percentage of observations in the node. Here, we see that Gender is most important covariate for predicting the performance of an IBT course participant as per the 2017 data.

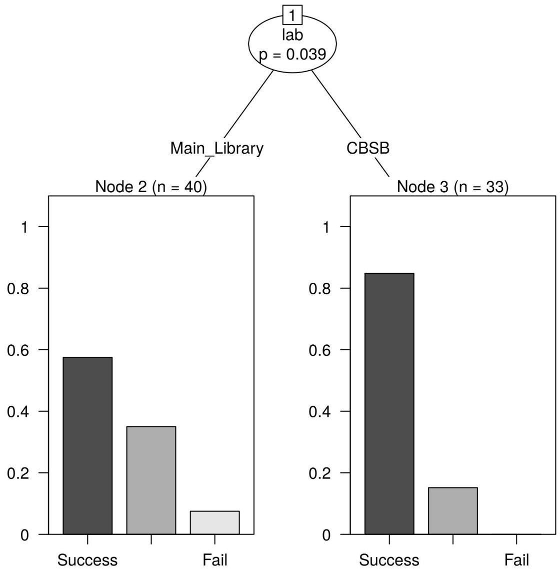

Figure SF6: **Conditional Inference Tree for Participants Performance in the IBT.** Here, we se that the physical classroom location is the most important covariate in predicting an IBT participant's performance

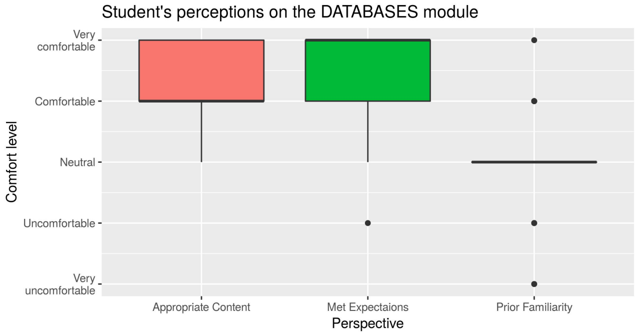

Figure SF7: IBT participants perceptions: Databases module

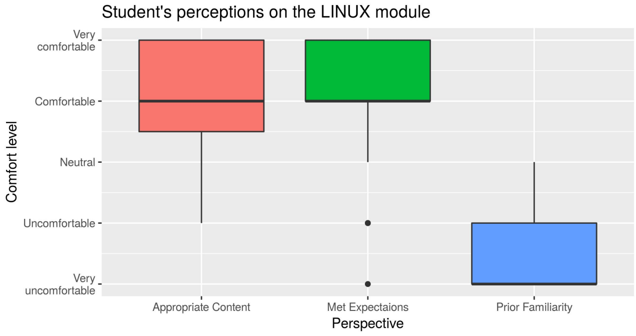

Figure SF8: IBT participants perceptions: Linux module

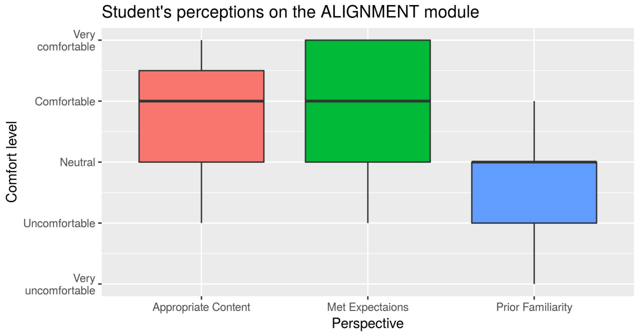

Figure SF9: IBT participants perceptions: Pair wise alignment module. For the prior familiarity, we used the same responses in producing figure SF10 , because participants were expected to have low familiarity with both types

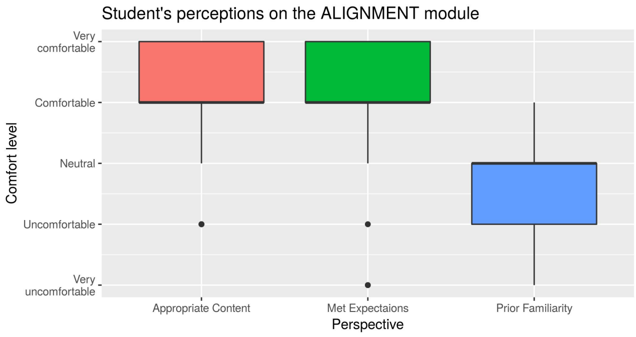

Figure SF10: IBT participants perceptions: multiple sequence alignment module. For the prior familiarity, we used the same responses in producing figure SF9 , because participants were expected to have low familiarity with both types

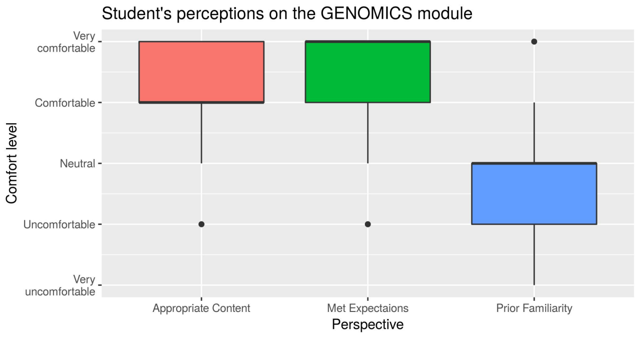

Figure SF11: IBT participants perceptions: Genomics module

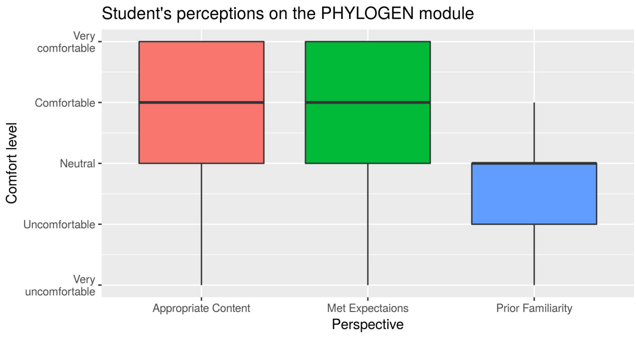

Figure SF12: IBT participants perceptions: phylogenetics module

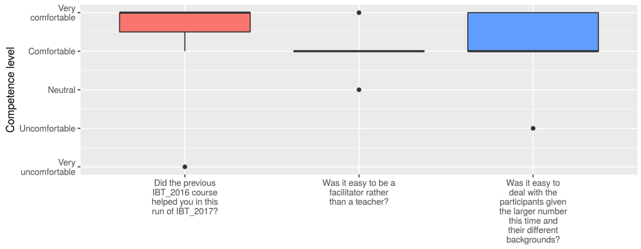

Figure SF13: TAs perspective: Evaluation of the teaching experience (n =7)

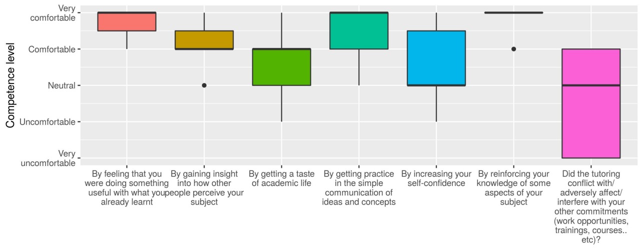

Figure SF14: TAs perspectives: Personal reflections (n=7)

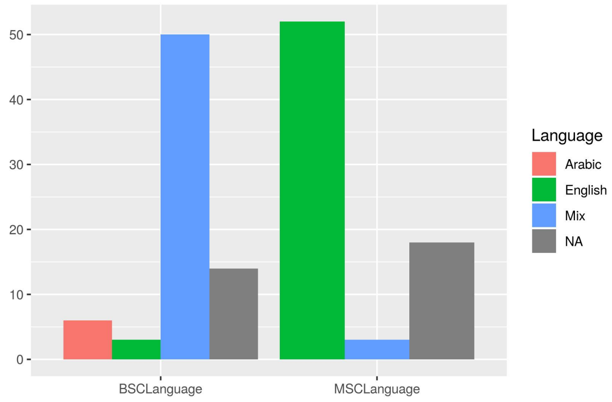

Figure SF15: Language of instruction of the IBT participants in their BSc and MSc education
