# Supplementary material for: Delivering blended bioinformatics training in resource-limited settings: a case study on the University of Khartoum H3ABioNet node
Source: Brief Bioinform. 2019 Feb 15;21(2):719–28. doi: 10.1093/bib/bbz004 (PMC7299290; doi:10.1093/bib/bbz004)
Supplement: Suppl_bbz004 [file suppl_bbz004.zip › SM4_Survey4_exit_survey_for_withdrawn_course_participants.pdf]

Exit survey: IBT_2017

Purpose: Results from this survey will be used to improve the quality of workshops delivered and for reporting purposes.

Therefore, please take time to fill this form in as fully as you can. Your feedback is highly recognized and appreciated

Please note that personal data will be made anonymous and will not affect your grade and status in any way. They would only be used to assure the integrity of the collected data

*Required

1. **Email address ***

2. **Full name: ***

3. **Have you attended any module session as part of this run of the IBT course? ***

   Mark only one oval.

   - No. I never attended any session *Skip to question 3.*
   - Yes: I only attended 1 session. only *Skip to question 3.*
   - Yes: I attended 2 or more sessions *Skip to question 4.*

Early withdrawal reasons

4. **Would you indicate the reason why you couldn't commit to the course? ***
   Kindly be reminded that your registration meant others were banned from attending because the seat was reserved.

Stop filling out this form.

Course reflections

5. **Where were you based?** *
Mark only one oval.

- ( ) CBSB lab
- ( ) Main library

6. **How would you rate your local classroom environment?** *
1 is very uncomfortable, 5 is very comfortable
Mark only one oval per row.

	1	2	3	4	5
Audio quality	( )	( )	( )	( )	( )
Internet access	( )	( )	( )	( )	( )
Air conditioning	( )	( )	( )	( )	( )
Accessibility to services (bathrooms & cafeteria)	( )	( )	( )	( )	( )
Allocated number of local Teaching Assistants	( )	( )	( )	( )	( )

7. **How many sessions have you attended from the course?** *
Mark only one oval.

- ( ) 4-8
- ( ) 9-12
- ( ) 13- or more
- ( ) I don't recall

8. **How many assignments have you submitted?** *
Also, kindly indicate if you attended any module, but didn't submit the corresponding assignments

____

9. **How many assessments have you submitted?** *

____

10. **Have you ever used Vula forum or chat?** *
Mark only one oval.

- ( ) Yes
- ( ) No
- ( ) Other: ______________________________

11. **To what extent was any of the following a reason for you to leave the course?** *

Mark only one oval per row.

	Very strong reason	Relatively a reason	Didn't have effect	Relatively not a reason	Not a reason at all
3 months is too long a period for a course					
Language of instruction was hard					
Language of instruction was too fast					
Lectures were overwhelming					
Modules did not meet my expectations					
Flow of course contents was not systematic					
I didn't have enough background information					
No pre-requisit materials to go through in advance (pre-class relevant material))					
Difficulty in using Vula account					
Difficulty in Assignments and assessments					
Inefficiency of support form the local TAs					
Unablity to interact with colleagues in your local class					
Personal reasons (work related)					
Personal reasons (family related)					
Technical reasons (Linux based PCs, ... etc)					

12. **What are your recommendations to improve any of the logistics or aspects of the course?** *

Powered by
Google Forms
