# Supplementary material for: Delivering blended bioinformatics training in resource-limited settings: a case study on the University of Khartoum H3ABioNet node
Source: Brief Bioinform. 2019 Feb 15;21(2):719–28. doi: 10.1093/bib/bbz004 (PMC7299290; doi:10.1093/bib/bbz004)
Supplement: Suppl_bbz004 [file suppl_bbz004.zip › SM2_Survey2_Mid_course.pdf]

Mid-course Assessment survey: IBT_2017

Purpose: Results from this local survey will be used to improve the quality of the IBT course delivered and for reporting purposes.

Please take 5-10 minutes to fill-in this form as fully as you can. Your feedback is highly recognized and appreciated.

Please note that personal data will be made anonymous and will not affect your grade and status in any way. They would mainly be used to assure the integrity of the collected data

*Required

1. **Email address** *

____

2. **Full name:** *

____

Educational background

3. **Highest educational degree obtained** *
Mark only one oval.
   - ( ) BSc
   - ( ) MSc
   - ( ) PhD
   - ( ) Other: ______________________

4. **University** *
What University awarded your highest degree?

____

5. **Faculty** *
What Faculty awarded your highest degree?

____

6. **Department**
What department within that faculty awarded your highest degree?

____

7. **Graduation Specialization** *
What was your specialization in your highest degree? Kindly select from the list below, or specify in the "Others" field if your specialization is not in the list:
Tick all that apply.

- [ ] Genetics and Molecular biology
- [ ] Biochemistry
- [ ] Other: ______

Current status

8. **Current position** *
Check all that apply
Tick all that apply.

- [ ] Graduate student (MSc or PhD)
- [ ] Researcher
- [ ] Lecturer
- [ ] Assistant professor
- [ ] Other

9. **Current affiliation** *
Where do you work now? (please put NA if not working now)

______

10. **Current bioinformatics research areas of interest** *
Tick all that apply.

- [ ] Database resources
- [ ] Sequence Alignment
- [ ] Comparative Genomics
- [ ] Genome variation
- [ ] Genome annotation
- [ ] Phylogenetics
- [ ] Proteomics and Structural Bioinformatics
- [ ] Other: ______

General Course evaluation

11. **Rate how comfortable you are with the following aspects of the course** *
Note: a rating of 5 is very comfortable, 1 is Not at all
Mark only one oval per row.

	1	2	3	4	5
Submission of assignments to Vula					
Asking/ Answering question in Vula forum/chat					
Networking with others from your classroom					
Networking with others from other classrooms					

12. **How would you rate your learning environment?** *
Note: a rating of 5 is very comfortable, 1 is Not at all
Mark only one oval per row.

	1	2	3	4	5
Audio quality					
Internet access					
Air conditioning					
Accessibility to services (bathrooms & cafeteria)					

Specific Course evaluation

13. **For "Module 1: Databases and Resources", rate each of the following items** *
Note: a rating of 5 is very comfortable, 1 is Not at all
Mark only one oval per row.

	1	2	3	4	5
The module met your expectations					
Content of the module is appropriate					
Assignments and assessments were relevant					
The instructor was communicating clearly					
The instructor was reponsive in the forum					
The local Teaching Assistants were supportive					

14. **For "Module 2: Linux", rate each of the following items** *
Note: a rating of 5 is very comfortable, 1 is Not at all
Mark only one oval per row.

	1	2	3	4	5
The module met your expectations					
Content of the module is appropriate					
Assignments and assessments were relevant					
The instructor was communicating clearly					
The instructor was reponsive in the forum					
The local Teaching Assistants were supportive					

15. **For "Module 3: Sequence alignment", rate each of the following items** *
Note: a rating of 5 is very comfortable, 1 is Not at all
Mark only one oval per row.

	1	2	3	4	5
The module met your expectations					
Content of the module is appropriate					
Assignments and assessments were relevant					
The instructor was communicating clearly					
The instructor was reponsive in the forum					
The local Teaching Assistants were supportive					

16. **What are your recommendations to improve any of the logistics or any aspect of the course?** *

Send me a copy of my responses.

Powered by
Google Forms
