# Supplementary material for: Delivering blended bioinformatics training in resource-limited settings: a case study on the University of Khartoum H3ABioNet node
Source: Brief Bioinform. 2019 Feb 15;21(2):719–28. doi: 10.1093/bib/bbz004 (PMC7299290; doi:10.1093/bib/bbz004)
Supplement: Suppl_bbz004 [file suppl_bbz004.zip › SM3_Survey3_End_course.pdf]

Final-course Assessment survey: IBT_2017

Purpose: Results from this survey will be used to improve the quality of workshops delivered and for reporting p
Therefore, please take time to fill this form in as fully as you can. Your feedback is highly recognized and appreciated

Please note that personal data will be anonymouised and will not affect your status in any way. They would mainly be used to assure the integrity of the collected data

*Required

1. **Email address** *

   ____

2. *Mark only one oval.*

   ◯ Option 1

3. **Full name:** *

   ____

Course evaluation

4. **For module 6: Phylogenetics, to what extent was:** *
   1 is very uncomfortable, 5 is very comfortable
   Mark only one oval per row.

	1	2	3	4	5
The module met your expectation	◯	◯	◯	◯	◯
Content of the modules given appropriate	◯	◯	◯	◯	◯
Assignments and assessments were relevant	◯	◯	◯	◯	◯
The instructor was speaking fast, or unclearly	◯	◯	◯	◯	◯
The instructor was reponsive in the forum	◯	◯	◯	◯	◯
The local Teaching Assistants were supportive	◯	◯	◯	◯	◯

5. **For module 5: Genomics, to what extent was** *
1 is very uncomfortable, 5 is very comfortable
Mark only one oval per row.

	1	2	3	4	5
The module met your expectation					
Content of the modules given appropriate					
Assignments and assessments were relevant					
The instructor was speaking fast, or unclearly					
The instructor was reponsive in the forum					
The local Teaching Assistants were supportive					

6. **For module 4: Multiple Sequence alignment, to what extent was:** *
1 is very uncomfortable, 5 is very comfortable
Mark only one oval per row.

	1	2	3	4	5
The module met your expectation					
Content of the modules given appropriate					
Assignments and assessments were relevant					
The instructor was speaking fast, or unclearly					
The instructor was reponsive in the forum					
The local Teaching Assistants were supportive					

General feedback

7. **What are your recommendations to improve any of the logistics or any aspect of the course?** *

8. **Briefly describe your experience in the IBT course.** *

9. **I hereby, would like to give permission and consent for my responses to be used as quotes about CBSB courses**
   Mark only one oval.

   - Yes
   - No

10. **Will you be confident to recommend the IBT course to others?** *
    Mark only one oval.

    - Yes
    - No
    - Maybe

11. **In your opinion, what of the following would further enhance your bioinformatics learning** *
    Tick all that apply.

    - Providing more advanced courses exploring some of the topics in more details
    - Having an opportunity for a face-to-face training with instructors
    - Having focused courses run in a shorter duration
    - Working in projects with bioinformatics components
    - Other: ______________________

12. **Do you think the topics of the modules were very basic or advanced?** *

    ____

13. **To what extent was the IBT 2017 comparable to other Bioinformatics workshops you have attended previously?** *
    Hint: 1 means not comparable at all, 5 means very similar
    Mark only one oval per row.

	1	2	3	4	5
Language wise					
course natrue of interaction (face-to-face)					
access to international community					
international recognition of the certificate					
content and scope					
pace of the course					
forming of new relations					
Duration					

14. **Rate how comfortable you are with the following aspects of the course** *
Copy from the exit survey!
Mark only one oval per row.

	1	2	3	4	5
Language of instruction					
Overall content of the taught modules					
Submission of assignments to Vula					
Asking/ Answering question in Vula forum/chat					
Networking with others from your classroom					
Networking with others from other locations					

15. **In the future which do you intend to attend more, Online courses or Face-to-face courses? Why?** *

16. **How would you rate your local classroom environment?** *
1 is very uncomfortable, 5 is very comfortable
Mark only one oval per row.

	1	2	3	4	5
Audio quality					
Internet access					
Air conditioning					
Accessibility to services (bathrooms & cafeteria)					
Support from the local staff (Faculty and Library Staff)					

Research Areas of interest

17. **What are the Bioinformatics research areas of interest that you wish to pursuit?**
We know we asked this before, but maybe you changed your mind with the new content introduced
Mark only one oval.

- Database resources
- Sequence Alignment
- Comparative Genomics
- Genome variation
- Genome annotation
- Phylogenetics
- Proteomics and Structural Bioinformatics
- Other: ______________________

Powered by
Google Forms
