# Supplementary material for: Delivering blended bioinformatics training in resource-limited settings: a case study on the University of Khartoum H3ABioNet node
Source: Brief Bioinform. 2019 Feb 15;21(2):719–28. doi: 10.1093/bib/bbz004 (PMC7299290; doi:10.1093/bib/bbz004)
Supplement: Suppl_bbz004 [file suppl_bbz004.zip › SM5_Survey5_Teaching_assistants.pdf]

Teaching Assistantship survey: IBT_2017

Results from this local survey will be used to improve the quality of the IBT course delivered and for reporting purposes, and make it as beneficial and fair to everyone taking part.

Please take 5-10 minutes to fill-in this form as fully as you can. Your feedback is highly recognized and appreciated.

Please note that personal data will be made anonymous and will not affect your status in any way. They would mainly be used to assure the integrity of the collected data

*Required

1. **Email address** *

2. **Full name:** *

3. **To what extent:** *
   Note: a rating of 5 is highly applicable, 1 is Not at all
   Mark only one oval per row.

	1	2	3	4	5
Was it easy to be a facilitator rather than a teacher?					
Did the previous IBT_2016 course helped you in this run of IBT_2017?					
Was it easy to deal with the participants given the larger number this time and their different backgrounds?					

4. **What do you think about the IBT core team activities (Staff training, Staff meeting, Mconf sessions)** *

5. **What do you think about our local activities (Meetings, Paper writing, dealing with web-based platforms)?** *

6. **Do you think that you benefited from being a tutor in any of the following ways?** *

Note: a rating of 5 is highly applicable, 1 is Not at all
Mark only one oval per row.

	1	2	3	4	5
By getting practice in the simple communication of ideas and concepts					
By reinforcing your knowledge of some aspects of your subject					
By gaining insight into how other people perceive your subject					
By increasing your self-confidence					
By feeling that you were doing something useful with what you already learnt					
Did the tutoring conflict with/adversely affect/interfere with your other commitments (work opportunities, trainings, courses.. etc)?					
By getting a taste of academic life					

7. **What did you like least about the tutoring and how can it be improved?**

8. **Do you think students tutoring will be in your career path? Yes/No. If yes – in what way?** *

9. **Have you any other comments? Are there any episodes which you will remember as particularly rewarding or disturbing?** *

10. **I hereby, would like to give permission for my responses to be used as quotes about tutoring (in the future runs of courses)** *
Mark only one oval.

- Yes
- No

Powered by
Google Forms
