# Supplementary material for: Delivering blended bioinformatics training in resource-limited settings: a case study on the University of Khartoum H3ABioNet node
Source: Brief Bioinform. 2019 Feb 15;21(2):719–28. doi: 10.1093/bib/bbz004 (PMC7299290; doi:10.1093/bib/bbz004)
Supplement: Suppl_bbz004 [file suppl_bbz004.zip › SM6_Survey6_follow_up_on_participants_after_9_months.pdf]

Alumni survey: IBT_2017

Purpose: Results from this survey will be used to improve the quality of workshops delivered and for reporting purposes. In addition, your feedback will help us in planning for an Intermediate Bioinformatics course based on the foundation of the IBT.

Therefore, kindly take time to fill this form in as fully as you can. It should not take more than 3-5 minutes, and your feedback will be highly recognized and appreciated.

Please note that personal data will be made anonymous and will not affect your status in any way. They would mainly be used to assure the integrity of the collected data

*Required

1. **Email address** *

2. **Full name:** *

At the time of taking the IBT..

3. **What was your academic position at the time of taking the IBT..?** *
   Tick all that apply.
   - [ ] Student (no formal position)
   - [ ] Junior staff (e.g. assistant lecturer/ lecturer or equivalent)
   - [ ] Middle staff (e.g. senior lecturer or equivalent)
   - [ ] Senior staff (e.g. Group leader / Professor / Head of Departent etc)

4. **What was your highest degree completed at that time?** *
   Mark only one oval.
   - ( ) BSc or equivalent
   - ( ) MSc or equivalent
   - ( ) PhD/ MD or equivalent
   - ( ) Prof. or equivalent

What about your current (or near future) position

5. **What is your academic position now? ***
   Tick all that apply.

   - [ ] Student (no formal position)
   - [ ] Junior staff (e.g. assistant lecturer/ lecturer or equivalent)
   - [ ] Middle staff (e.g. senior lecturer or equivalent)
   - [ ] Senior staff (e.g. Group leader / Professor / Head of Departent etc)

6. **What is your highest completed degree? ***
   Mark only one oval.

   - ( ) BSc or equivalent
   - ( ) MSc or equivalent
   - ( ) PhD/ MD or equivalent
   - ( ) Prof. or equivalent

General feedback

7. **In what way(s) did the IBT experience benefit you?**
   Tick all that apply.

   - [ ] Refine my ongoing research question
   - [ ] New job offerings in Sudan (TA, researcher, .. etc)
   - [ ] New position abroad (PhD offers, other jobs.. etc)
   - [ ] Open new dimension in research
   - [ ] Other: ____________________

8. **Are there any interesting in-between steps you would like to tell us about? ***

   ____

9. **I hereby, would like to give permission and consent for my responses to be used as quotes about CBSB courses**
   Mark only one oval.

   - ( ) Yes
   - ( ) No

Powered by
Google Forms
