# Supplementary material for: Delivering blended bioinformatics training in resource-limited settings: a case study on the University of Khartoum H3ABioNet node
Source: Brief Bioinform. 2019 Feb 15;21(2):719–28. doi: 10.1093/bib/bbz004 (PMC7299290; doi:10.1093/bib/bbz004)
Supplement: Suppl_bbz004 [file suppl_bbz004.zip › SM1_Survey1_Start.pdf]

Assessment survey: IBT_2017

Background Assessment Survey for IBT_2017 Batch

*Required

1. **Email address** *

2. **Full name:** *

3. **Educational background** *
   For example, molecular biology, pharmacy, botany, zoology, ...etc

4. **Highest educational degree obtained** *
   Mark only one oval.
   - BSc
   - MSc
   - PhD

5. **The institute awarding the highest degree** *
   For example, Institute of Endemic disease, University of Khartoum --- Department of zoology, Faculty of Science, University of Khartoum --- Faculty of Pharmacy, University of Khartoum, ...etc

6. **Main language of instruction for undergraduate studies** *
   Mark only one oval.
   - English
   - Arabic
   - Other:

7. **Main language of instruction for postgraduate studies** *
Mark only one oval.

- English
- Arabic
- Other: ____________

8. **Current affiliation** *

____

9. **Current research areas of interest or projects** *

____

10. **How do you describe your familiarity with the following:** *
Mark only one oval per row.

	Expert	Very familiar	Familiar	Unfamiliar	Totally unfamiliar
Public databases (eg NCBI, EBI, ..)					
Linux					
Programming					
Genomics					
Sequence alignment theory					
Molecular evolution and phylogenitics					
Structural bioinformatics & Proteomics					

11. **What previous Bioinformatics courses or workshops have you attended?** *
Also, state when, where you took them and the duration of the workshop/ training. For example, Course xxxxx, University of yyyyy, country zzzzz, from 5/3/2017 to 2/4/2017

____
____
____
____
____

12. **What are your main expectations out of this course? ***

____

____

____

____

____

13. **How did you hear of this course? ***
Tick all that apply.

- [ ] Email
- [ ] Facebook/ Twitter
- [ ] Word of a friend
- [ ] Supervisor/Mentor
- [ ] Other: ______________________________

Powered by
Google Forms
